# Gene Entropy-Fractal Dimension Informatics with Application to Mouse-Human Translational Medicine

**DOI:** 10.1155/2013/582358

**Published:** 2013-03-17

**Authors:** T. Holden, E. Cheung, S. Dehipawala, J. Ye, G. Tremberger, D. Lieberman, T. Cheung

**Affiliations:** Queensborough Community College of CUNY, 222-05 56th Avenve Bayside, NY 11364, USA

## Abstract

DNA informatics represented by Shannon entropy and fractal dimension have been used to form 2D maps of related genes in various mammals. The distance between points on these maps for corresponding mRNA sequences in different species is used to study evolution. By quantifying the similarity of genes between species, this distance might be indicated when studies on one species (mouse) would tend to be valid in the other (human). The hypothesis that a small distance from mouse to human could facilitate mouse to human translational medicine success is supported by the studied ESR-1, LMNA, Myc, and RNF4 sequences. ID1 and PLCZ1 have larger separation. The collinearity of displacement vectors is further analyzed with a regression model, and the ID1 result suggests a mouse-chimp-human translational medicine approach. Further inference was found in the tumor suppression gene, p53, with a new hypothesis of including the bovine PKM2 pathways for targeting the glycolysis preference in many types of cancerous cells, consistent with quantum metabolism models. The distance between mRNA and protein coding CDS is proposed as a measure of the pressure associated with noncoding processes. The Y-chromosome DYS14 in fetal micro chimerism that could offer protection from Alzheimer's disease is given as an example.

## 1. Introduction

When a nucleotide in a DNA sequence is different from the preceding nucleotide, this is defined as a nucleotide fluctuation. The nucleotide fluctuations of a DNA sequence can be studied as a series using the nucleotide atomic number of the nucleotide A, T, C, and G. A recent study on such fluctuation in the FOXP2 gene has been reported [1]. The fractal dimension and Shannon entropy was found to have a negative correlation (*R* ^2^ ~ 0.85  *N* = 12) for the FOXP2 regulated accelerated conserved noncoding sequences in human fetal brain. This paper uses a 2D mapping of the Shannon entropy and fractal dimension to determine displacement vectors, which could serve as a marker for the evolutionary differences between mouse and human DNA in clinically important gene sequences. The hypothesis that displacement vectors having small separation would facilitate the mouse to human translational medicine success would be testable with gene therapy cases. The selected gene candidates in this report are based on new discoveries reported in and around September 2012. The ESR1 neuronal estrogen receptor was reported by Rockefeller University to be a single “mommy” gene such that malfunction deletion would suppress motherhood behavior [2]. Successful control of Hutchinson-Gilford progeria syndrome in children by correcting the mutated LMNA lamin A protein was reported by Harvard Medical School [3]. The Myc myelocytomatosis oncogene was reported by US National Institutes of Health to be a universal amplifier for cancer already turned on by another process [4]. The RNF4, RING finger protein 4 with zinc finger motif, was reported by UK Dundee University to be necessary for human response to DNA damage [5]. The ID1, a DNA-binding protein inhibitor, associated with aggressive nonstandard breast cancer cells could be controlled by cannabidiol in cannabis [6]. The PLCZ1, phospholipase C Zeta 1, was reported to be delivered by the sperm to control egg activation [7]. Calibration based on 16S rRNA (human and mouse) enables a relative measure of the evolutionary pressure of the above genes between human and mouse. The HAR1 sequence with 118-bp, is the fastest evolving human sequence as compared to the chimp. It contains 18 point substitutions occurring over a span of 5 million years when comparing the human to the chimpanzee. However, the same 118-bp region only contains two-point substitutions over a span of 300 million years when comparing the chimpanzee to the chicken [8]. The inclusion of HAR1 in the calibration should set an upper limit for the displacement vector magnitude. 

## 2. Materials and Methods

The data used in this study was downloaded from Genbank and the accession information is listed [9–18]. The HAR1 human and chimp sequences were downloaded with information from [8]. 

A sequence with a relatively low nucleotide variety would have low Shannon entropy (more constraint) in terms of the set of 16 possible dinucleotide pairs. A sequence's entropy can be computed as the sum of (*p*_*i*_)∗log⁡(*p*_*i*_) over all states *i*, and the probability *p*_*i*_ can be obtained from the empirical histogram of the 16 di-nucleotide-pairs. The maximum entropy is 4 binary bits per pair for 16 possibilities (2^4^). For mononucleotide consideration, the maximum entropy is two bits per mono with four possibilities (2^2^). The mononucleotide entropy is correlated to dinucleotide entropy *R* ^2^ > 0.9 for all studied sequences in the project.

Fractal dimension analysis on data series can be used in the study of correlated randomness. Among the various fractal dimension methods, the Higuchi fractal method is well suited for studying fluctuation [19]. The spatial intensity (Int) series with equal intervals is be used to generate a difference series (Int(*j*) − Int(*i*)) for different lags (*j* − *i*) in the spatial variable. The nonnormalized apparent length of the spatial series curve is simply *L*(*k*) = Σ | Int(*j*) − Int(*i*)| for all (*j* − *i*) pairs that equal to *k*. The number of terms in a *k*-series varies, and normalization must be used to get the series length. If the Int(*i*) is a fractal function, then the log⁡(*L*(*k*)) versus log⁡(1/*k*) should be a straight line with the slope equal to the fractal dimension. Higuchi incorporated a calibration division step such that the maximum theoretical value is calibrated to the topological value of 2. The detailed calculation is given in the literature [19]. The Higuchi fractal algorithm used in this project was calibrated with the Weierstrass function. This function has the form *W*(*x*) = Σ *a* ^−*nh*^cos⁡(2*πa* ^*n*^ *x*) for *n* = 0, 1,2, 3,…. The fractal dimension of the Weierstrass function is given by (2 − *h*), where *h* takes on an arbitrary value between zero and one.

Although the Higuchi method was originally developed for time series data, Fractal dimension analysis is an established method to analyze DNA sequences and other finite progressions [20]. By comparing the fractal dimension for a concatenated infinite sequence of known fractal dimension, we obtain results similar to those shown in Figure 8 of [21]. For the lengths of sequences analyzed in this paper, the error is about 1% or less, corresponding to about one fifth of the variation in fractal dimension seen in this paper. Thus, we conclude that the current analysis is justified for these sequences.

## 3. Results of Fractal Analysis

**Figure 1:**
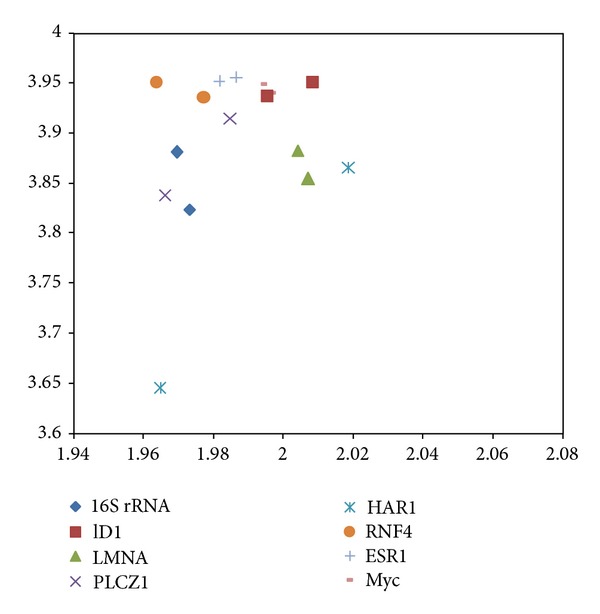
The mRNA 2D map of the studied mouse-human pairs. The *y*-axis represents dinucleotide entropy in bits per symbol, and *x*-axis presents the fractal dimension. 16S rRNA (diamond), ID1 (square), PLCZ1 (cross), RNF4 (circle), ESR1 (plus), and Myc (bar) have lower fractal dimensions for human. The LMNA (triangle) and HAR1 have higher fractal dimension for human.

**Figure 2:**
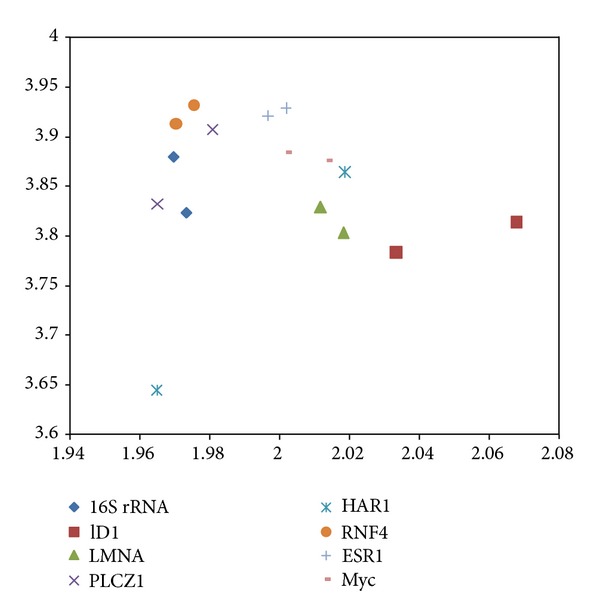
The protein coding CDS 2D map of the studied mouse-human pairs. The *y*-axis represents di-nucleotide entropy in bits per symbol, and *x*-axis presents the fractal dimension. 16S rRNA (diamond), ID1 (square), PLCZ1 (cross), ESR1 (plus), and Myc (bar), have lower fractal dimension for human. LMNA (triangle), HAR1 (star), and RNF4 (circle) have higher fractal dimension for human.

**Figure 3:**
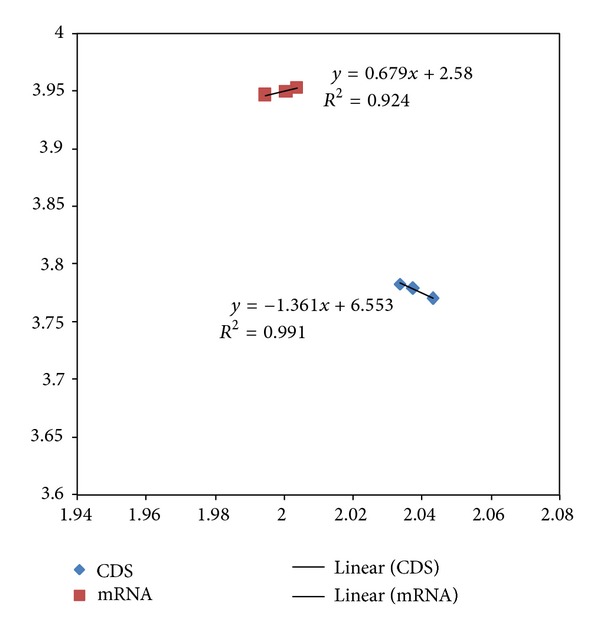
The regression model of human ID1 variant1, human ID1 variant2, and chimp ID1. The *y*-axis represents di-nucleotide entropy in bits per symbol, and *x*-axis presents the fractal dimension. The graph scale is kept identical to that of Figures 1 and 2 for easy comparison. The CDS sequence (diamond) regression has *R* ^2^ of 0.991 and an adjusted *R* ^2^ of 0.983 (the chimp has the highest fractal dimension). The mRNA (square) sequence regression has *R* ^2^ of 0.924 (the chimp is in the middle among the three data points).

The mRNA and protein coding CDS 2D maps of entropy and fractal dimension of the studied mouse-human pairs are shown below in Figures 1 and 2, respectively. The mRNA human sequences except LMNA and HAR1 show lower fractal dimension as compared to the mouse counterparts. The CDS human sequences except LMNA, HAR1, and RNF4 show lower fractal dimension as compared to the mouse counterparts. Furthermore, the separation from one point to another could be represented by a displacement vector. A regression model is applicable for ID1 human variant 1, human variant 2, and chimp given the collinearity of the displacement vectors. The ID1 regression result is displayed in Figure 3. The graph scale is identical to that of Figures 1 and 2 for easy comparison. The *x*-axis fractal dimension should not be interpreted as the independent variable.

## 4. Discussion

The mouse to human difference is represented by the coordinate separation in Figure 1 (mRNA sequences) and Figure 2 (CDS sequences). HAR1 has the most separation in terms of coordinates in Figure 1, consistent with the labeling of the most accelerated region, given 18 point mutation from chimp to human in 118-bp. The HAR1 mouse counterpart is close to HAR1 chimp counterpart and has a fractal dimension of 1.945 and 3.657 bits per symbol (not displayed). The CDS map in Figure 2 shows ID1 having the most separation, followed by PLCZ1. BLAST comparison of mouse versus human results show *E*-value of zero for PLCZ1, suggesting that the entropy-fractal dimension 2D map can have a finer resolution. A large coordinate separation would be expected to represent very different sets of regulatory pathways from mouse to human. When comparing Figure 1 with Figure 2, the spreading of CDS data points as compared to the mRNA data points is dominated by ID1 coordinate change. For example, the coordinate change of CDS-ID1 from mouse to human would be comparable to the HAR1 separation representing an evolutionary aspect from chimp to human. Furthermore, as collinearity in displacement vectors could be represented by regression, the result of the coordinate changes in the CDS map of Figure 2 from that the mRNA map of Figure 1 increases the collinearity of the displacement vectors. For example, for ID1 in human variant 1, human variant 2, and chimp, the coordinate changes from mRNA to CDS have resulted in an increasing *R* ^2^ from 0.93 (mRNA) to 0.99 (CDS) as displayed in Figure 3.

**Figure 4:**
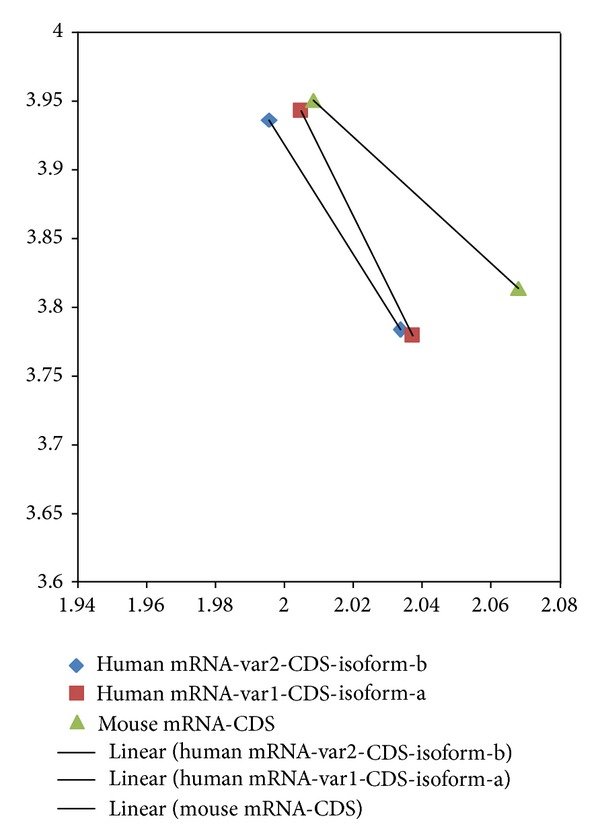
Displacement vector from mRNA to CDS for human ID1, and mouse ID1. The *y*-axis represents di-nucleotide entropy in bits per symbol and *x*-axis presents the fractal dimension. The separation or distance is shown as the length of the displayed line and the direction is from mRNA coordinates (upper diamond, upper square and upper triangle) to CDS coordinates (lower diamond, lower square and lower triangle).

**Figure 5:**
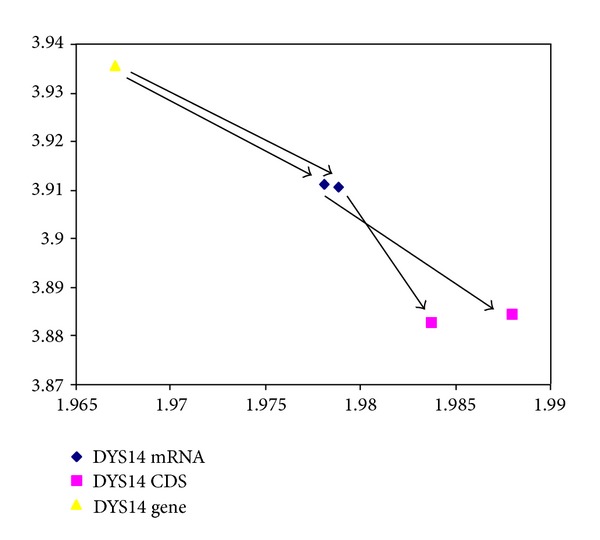
Entropy-fractal dimension map for Y-chromosome DYS14 Gene, mRNA, and CDS. The *y*-axis represents di-nucleotide entropy in bits per symbol, and *x*-axis presents the fractal dimension. The DYS14 gene (triangle) has the lowest fractal dimension, and the DYS14 CDS variant-1 and variant-2 (squares) are of higher fractal dimension, displayed as two data points in the lower right corner. DYS14 mRNA variant-1 and variant-2 (diamonds) have intermediate fractal dimension in comparison. The arrows represent the displacement vectors.

If one defines evolutionary pressure as the cause of species transformation, then CDS pressure could be defined as the cause of informatics transformation from mRNA to CDS and, correspondingly, mRNA pressure be defined as the cause of informatics transformation from gene to mRNA. A displacement vector in Figure 4 (denoted by a line) would represent the mRNA pressure in ID1 for human, and mouse also. A displacement vector in the 2D map formed in comparing Figures 1 and 2 would represent the CDS pressure. The collinearity of displacement vectors modeled as regression would represent the evolutionary pressure from chimp to human. A vector carries two pieces of information. A displacement vector carries separation or distance or magnitude information and directionality information such as from mRNA to CDS and chimp to human. A displacement vector analysis of  Y-chromosome DYS14 in fetal microchimerism was performed, and the result is displayed in Figure 5 where the selection of higher fractal dimension in mRNA pressure and CDS pressure is clearly demonstrated. The retention of DYS14 in a mother's brain was also reported to be consistent with protection for Alzheimer's disease for mothers who had sons [22].

**Figure 6:**
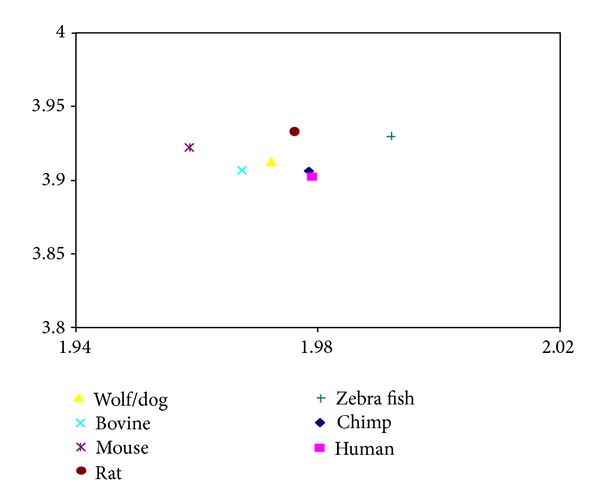
The protein coding CDS 2D map of the studied p53 sequences. The *y*-axis represents di-nucleotide entropy in bits per symbol, and *x*-axis presents the fractal dimension. The studied sequences included wolf/dog (triangle), bovine (cross), mouse (star), rat (circle), zebra fish (plus), chimp (diamond), and human (square).

**Figure 7:**
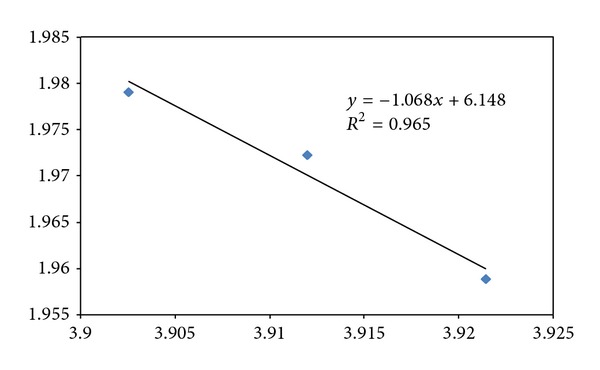
Entropy-fractal dimension for p53 CDS. The *x*-axis represents di-nucleotide entropy in bits per symbol, and *y*-axis presents the fractal dimension. Human has the highest fractal dimension, followed by wolf/dog, and mouse with the lowest fractal dimension.

**Figure 8:**
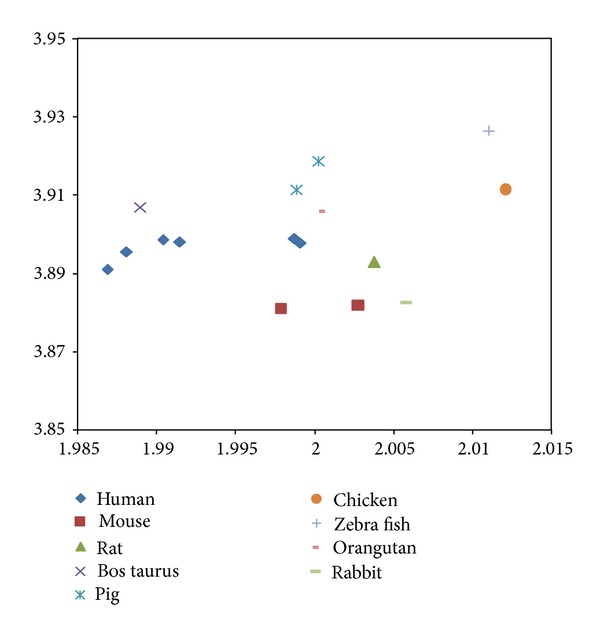
Entropy-fractal dimension for PKM2 CDS. The *y*-axis represents di-nucleotide entropy in bits per symbol, and *x*-axis presents the fractal dimension. The PKM2 of human (diamond, with 7 variants, gene no. 5315), mouse (square, with 2 variants, gene no. 18746), rat (triangle, gene no. 25630), bos Taurus (cross, gene no. 512571), pig (star, gene no. 100158154), chicken (circle, gene no. 396456), zebrafish (plus, gene no. 335817), orangutan (shorter bar, gene no. 100174114), and rabbit (longer bar, gene no. 100008676) are displayed.

A nucleotide sequence carries the informatics needed for a cell to live. A cell would continue to access the informatics throughout its lifetime. Average and standard deviation cannot represent the fluctuation or ordering of the nucleotides. Shannon entropy is a measure of the information content and fractal dimension could be interpreted as a measure of information order. In analogy to the Gas Law where pressure would be the cause of a temperature change given volume content, a displacement vector in the 2D map could be used as a marker for a pressure that would cause a fractal dimension change. Given the relatively large separation of ID1 as compared to the other studied sequences in Figure 2, a mouse-chimp-human approach would have supporting evidence. The data of other animals' ID1 sequences is shown in Figure 6, and using a mouse-monkey-human approach seems justified as well. Similarly, the Figure 7 CDS 2D map for the p-53 gene, known for its role in tumor suppression [23], would suggest a mouse-dog-human approach also to be valid. The collinearity represented by a regression gives an *R* ^2^ of 0.96, with adjusted *R* ^2^ ~ 0.93 (Figure 7). Recent advance in quantum metabolism modeling provides supporting evidence of natural section pressure on glycolysis preference over oxidative phosphorylation in cancerous environment [24]. The discovery of PKM2 dimeric form in elevated levels in many cancers has echoed the Warburg Effect in oncology and explained the rapid glycolysis [25]. The PKM2 evolutionary paths can be visualized in an entropy-fractal dimension 2D map (Figure 8). Targeting the PKM2 pathways could be a possible cancer therapy in the standard human-mouse model. The human-bovine (Bos Taurus) hypothesis could be a supplemental approach, especially for those conditions with lower fractal dimension value sequences among the seven PKM2 variants in human. The entropy-fractal dimension 2D map is a very sensitive tool for comparative analysis. An analogy would be a Fabry-Perot interferometer for resolving wavelengths given that the interference order is already selected. Translational medicine based on genetics would benefit from the entropy-fractal dimension 2D map analysis in the selection of a species model.

Other fractal analysis results with the aim of translational medicine application have been reported. The H1N1 virus hemagglutinin (HA) sequences from various strains have been classified with correlation matrix fractal dimension values ranging from 2.29 to 2.32 in using a DNA representation via the Voss indicator function [20, 26]. The multi-fractal property of myeloma multiple TET2 mRNA Variant1 and Variant2 has been shown to converge to 1.26 in fractal dimension [27]. In fact, such DNA representation has been applied to generate DNA walk patterns with wavelet analysis that reveals hidden symmetries [28, 29]. On the broader chromosome level, it was reported that the chromosome-3 in Caenorhabditis elegans has coding regions averaging 1.306 and noncoding regions averaging 1.298 in fractal dimension values [30]. The fundamental computer science string representation for DNA sequences has also been studied. Assigning binary strings such that A = (00), T = (11), C = (01), and G = (10) have been used for the study of olfactory receptor OR1D2 sequences in human, chimp, and mouse [31]. Other popular DNA representation schemes can be found in a recent computer science review where the relative strengths of several assignment schemes were compared. For example, the Galois indicator sequence where A = 0, T = 2, C = 1, and G = 3 would work well in exon detection [32]. Regardless of the DNA representation scheme, the complexity of a sequence would be revealed by fractal analysis.

A new hypothesis that high fractal dimension sequences may be top level regulators (transcription factors) recently discussed in the ENCODE project would deserve further investigation [33]. Other hypotheses, although not the main concern in translational medicine, could include high fractal dimension sequence as regulator for bioelectricity in microbes [34], optimal fractal dimension sequence for the photosynthesis genes involving quantum transport [35], and predicted entanglement process [36, 37].

## 5. Conclusions

 The DNA gene sequence informatics represented by Shannon entropy and fractal dimension have been used to form 2D maps, and coordinate changes have been used in a displacement vector formulation for the studying of evolution with directionality. Although fractal dimension only mathematically applies to infinite fractal series, we found the error introduced by the finite size of our DNA sequences to be less than one fifth of the observed variation, thus justifying our analysis from a mathematical perspective. The hypothesis that small displacement vector from mouse to human could facilitate mouse to human translational medicine success has received support from the studied ESR-1, LMNA, Myc, and RNF4 in terms of their CDS and mRNA sequences. The collinearity of displacement vectors is further analyzed with a regression model, and the ID1 result suggests a mouse-chimp-human translational medicine approach. Other systems were studied with similar results, including the tumor suppression p53 within a mouse-wolf(dog)-human framework, leading to a new hypothesis of including the bovine PKM2 pathways for targeting the glycolysis preference in many types of cancerous cells, thus supplementing quantum metabolism studies as well. The displacement vector from mRNA coordinates to protein coding CDS coordinates could be a measure of the CDS pressure associated with non-coding process. The Y-chromosome DYS14 in fetal microchimerism is given as an example that CDS pressure, as well as mRNA pressure from gene to mRNA, would result in a higher fractal dimension sequence. A new hypothesis that high fractal dimension sequences could be top level transcription factors recently discussed in the ENCODE project deserves further investigation.
